# Nitrate and bromate removal by autotrophic and heterotrophic denitrification processes: batch experiments

**DOI:** 10.1186/2052-336X-11-27

**Published:** 2013-12-19

**Authors:** Sevgi Demirel, Ibrahim Bayhan

**Affiliations:** 1 Environmental Engineering Department, Nigde University, Nigde, Turkey; 2 Environmental Health Branch, Sanliurfa Provincial Directorate of Health, Sanliurfa, Turkey

**Keywords:** Nitrate, Bromate, Autotrophic denitrification, Heterotrophic denitrification, Batch reactor

## Abstract

The effects of various parameters on bromate reduction were tested using lab-scale batch reactors with sulfur based autotrophic and methanol based heterotrophic denitrification processes. The initial bromate (BrO_3_^–^) concentration of 100 and 500 μg/L was completely reduced and bromide (Br^-^) was produced stoichiometrically from bromate in all batch reactors. In all experiments, nitrate was completely reduced to below detection limit. Kinetic studies showed that the sulfur-based autotrophic nitrate reduction rate increased with increasing initial nitrate concentration. At stoichiometrically sufficient methanol concentration as an external carbon source, nitrate and bromate were reduced to below US EPA drinking water limits in heterotrophic denitrification conditions. The methanol was completely depleted at the end of the heterotrophic operation conditions.

## Introduction

Bromate is a disinfection by-product produced from bromide (Br^-^) contained in water when ozonation is applied in the drinking water [1]. Bromate is a suspected genotoxic carcinogen and has been shown that it causes renal tumors in rats and mice [2]. A maximum allowed contaminant level of bromate is 10 μg/L by United States Environmental Protection Agency (US EPA). Bromate cannot be removed by traditional water treatment methods such as filtration, chlorination or lime softening [3].

Nitrate has become a problem in many part of the world as well as in Turkey. The US EPA has set the maximum contaminant levels of 10 mg NO_3_^_^N L^-1^ (nitrate) for drinking water [4]. Reverse osmosis, electrodialysis and ion exchange are the physico-chemical methods used for treatment of nitrate from drinking waters [5,6]. The main disadvantages of these methods are high operation and maintenance cost, generation of secondary waste brines. As an alternative, biological denitrification, either autotrophic or heterotrophic has been proposed separately for nitrate and/or bromate removal [7,8].

Sulfur-based autotrophic denitrification is an effective alternative due to low cost and availability of elemental sulfur [9]. It is insoluble and provides a slow release supply of electrons on demand, offering advantages of low maintenance.

In this process, the elemental sulfur acts as an electron donor, and nitrate serves as an electron acceptor. Hence, when nitrate is reduced to nitrogen gas, sulfur is oxidized to sulfate (reaction (1)).

(1) $$\begin{array}{l} NO_{3^{‒}}+1.10S^{0}+0.4CO_{2}+0.76H_{2}O\\ \;+0.08NH_{4^{+}}\to 0.08C_{5}H_{7}O_{2}N+1.10SO_{4^{2‒}}\\ \;+0.5N_{2}+1.28H^{+} \end{array}$$

On the other hand, heterotrophic denitrifying bacteria need an organic carbon source for respiration and growth. A wide variety of organic carbon sources have been used such as methanol, ethanol, glucose, acetate etc. If methanol is used as a carbon source, the stoichiometric relationships are written as follows (reaction (2)).

(2) $$\begin{array}{l} NO_{3^{‒}}+1.08CH_{3}\mathrm{OH}\\ \;+0.24H_{2}CO_{3}\to 0.056C_{5}H_{7}NO_{2}+0.47N_{2}\\ \;+1.68H_{2}O+\mathrm{HC}O_{3^{‒}} \end{array}$$

The aim of presented experiment was to evaluate the feasibility of the denitrification concept to the removal of a micropollutant such a bromate (in the μg/L range) from contaminated water, containing nitrate at much higher concentration (in the mg/L range) using batch reactors. The process has monitored for by products and performance of the autotrophic and heterotrophic denitrification processes was investigated in a series of experiments operated under different initial bromate, nitrate and methanol (as an organic carbon source) concentrations.

## Methods

### Sulfur-based autotrophic batch experiments

The sulfur-based autotrophic denitrification was tested in 150 mL serum bottles at 30°C, filled with medium supplemented with 50 mg/L K_2_HPO_4_. The batch reactors were consisted of 3 g sulfur and limestone particles. Batch experiments were carried out in three different nitrate concentrations (25, 50 and 75 mg/L) and two different bromate concentrations (100 and 500 μg/L) (Table 1). Then, the serum bottles were inoculated with the 5 mL (130 ±18 mg VSS/L medium) of denitrifying activated sludge which obtained from the first anoxic tank of a five stage Bardenpho process located in Harran University Campus (Sanliurfa, Turkey).

**Table 1:** Operational conditions in the sulfur-based batch reactors

**Batch Reactors**	**BrO**_**3**_^**- **^**(μg/L)**	**NO**_**3**_**-N (mg/L)**	**K**_**2**_**HPO**_**4**_
R1	100	25	50
R2	100	50	50
R3	100	75	50
R4	500	25	50
R5	500	50	50
R6	500	75	50

### Methanol-based heterotrophic batch experiments

Similar to autotrophic conditions, the heterotrophic denitrification was tested with same K_2_HPO_4_, NO_3_^-^, bromate and biomass concentrations (Table 2). In order to obtain heterotrophic denitrification conditions, methanol was added to the medium at two different concentrations. All the serum bottles were flushed with N_2_ gas to ensure anoxic conditions. All the serum bottles were sparged with N_2_ gas for 20 minutes to ensure the removal of dissolved oxygen as Sahinkaya < Dursun [10] reported.

**Table 2:** Operational conditions in the heterotrophic batch reactors

**Batch Reactors**	**BrO**_**3**_^**- **^**(μg/L)**	**NO**_**3**_**-N (mg/L)**	Metanol (mg/L)*	**K**_**2**_**HPO**_**4**_
R1	100	45	50 (19.7)	50
R2	500	45	50 (19.7)	50
R3	100	45	115 (45.3)	50
R4	500	45	115 (45.3)	50

*Values in parenthesis shows the methanol concentrations as DOC.

### Sampling and analytical techniques

Samples for analysis were collected for the measurement of BrO_3_^–^, Br^–^, NO_3_^–^, NO_2_^–^, dissolved organic carbon (DOC), sulfate, pH, and alkalinity. All samples were filtered over a 0.45 *μ*m-pore-size sterile filter. NO_3_^-^, NO_2_^-^ and SO_4_^2-^ concentrations were determined by ion chromatography (Schimadzu, Prominence HIC-NS, IC-A3 column). BrO_3_^–^ and Br^–^ were measured by ion chromotography (DIONEX-ICS 3000 with AS19 column, bromate detection limit of 3 μg/L). DOC was measured by TOC analyzer (TOC V_CPH_, Shimadzu, Japan).

## Results and discussion

### Performance of autotrophic denitrification process

In order to examine denitrification performance with initial different nitrate and bromate concentrations, 6 batch tests were performed under autotrophic conditions. In these batch experiments, the elemental sulfur acts as an electron donor and nitrate serves as an electron acceptor (reaction (1)). Hence, when nitrate is reduced to nitrogen gas, sulfur is oxidized to sulfate (reaction (1)).

**Figure 1:**
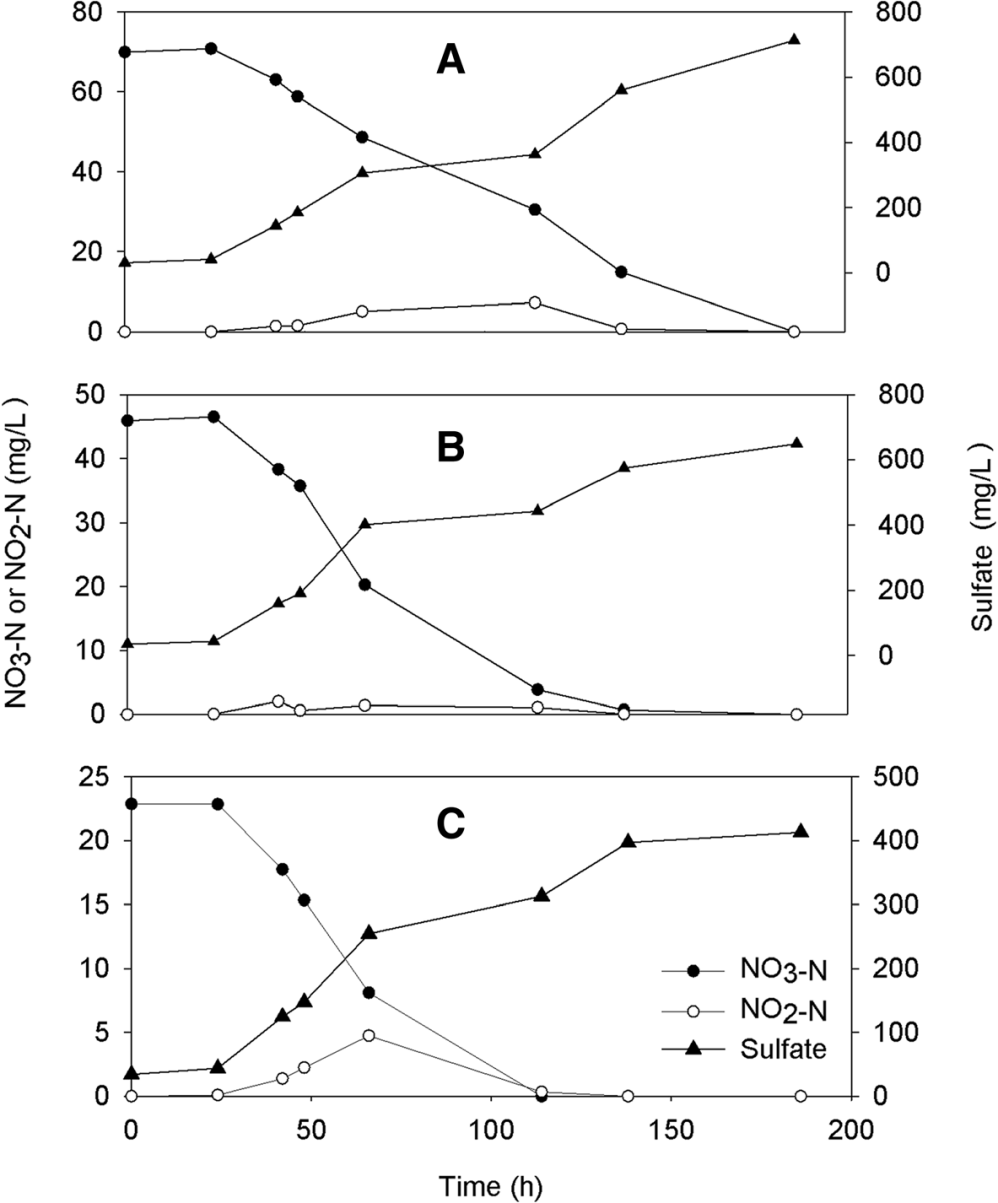
**The variations of NO**_**3**_^**-**^**-N, NO**_**2**_^**-**^**-N and sulfate concentrations in batch experiments at different initial NO**_**3**_^**-**^**-N concentrations [100 μg/L BrO**_**3**_^**-**^**]. (A)** 75 mg/L NO_3_^-^-N; **(B)** 50 mg/L NO_3_^-^-N; **(C)** 25 mg/L NO_3_^-^-N.

The batch reactors were operated under autotrophic conditions at two concentrations of bromate (100 and 500 μg/L) and three concentrations of nitrate (25, 50 and 75 mg/L). It was observed that nitrate concentration decreased after inoculation of denitrifiers to the batch reactors. Nitrite was detected as an intermediate and maximum NO_2_^-^-N concentration was reached 7.3 ± 3.5 mg/L during bromate and nitrate removal. NO_2_^-^-N was decreased rapidly after 100 hrs which indicates that denitrification occurred within the system (Figure 1).

The reduction rates of NO_3_^-^-N at various initial NO_3_^-^-N and BrO_3_^–^ concentrations were studied in batch serum bottles. The results of batch assays were summarized at Table 3. The theoretical sulfate productions calculated according to reaction (1) assuming complete denitrification were 189, 377 and 578 mg/L for 25, 50 and 75 mg/L nitrate respectively. The measured sulfate production was higher than the theoretically calculated values, which should be due to the input of oxygen during sampling.

**Table 3:** Denitrification rates for sulfur-based autotrophic denitrification

**BrO**_**3**_^**- **^**(μg/L)**	**NO**_**3**_**-N (mg/L)**	**Denitrification rates (mg NO**_**3**_**-N/L.day)**	**Maximum Nitrit Accumulation (mg NO**_**2**_**-N/L)**	**SO**_**4**_^**- **^**Production (mg/L)**
100	25	5.26	4.7 ± 0.9	412
	50	8.69	1.4 ± 2.3	649
	75	11.26	7.3 ± 3.5	712
500	25	4.63	2.3 ± 1.1	372
	50	8.27	4.9 ± 2.1	458
	75	9.67	5.0 ± 2.3	749

**Figure 2:**
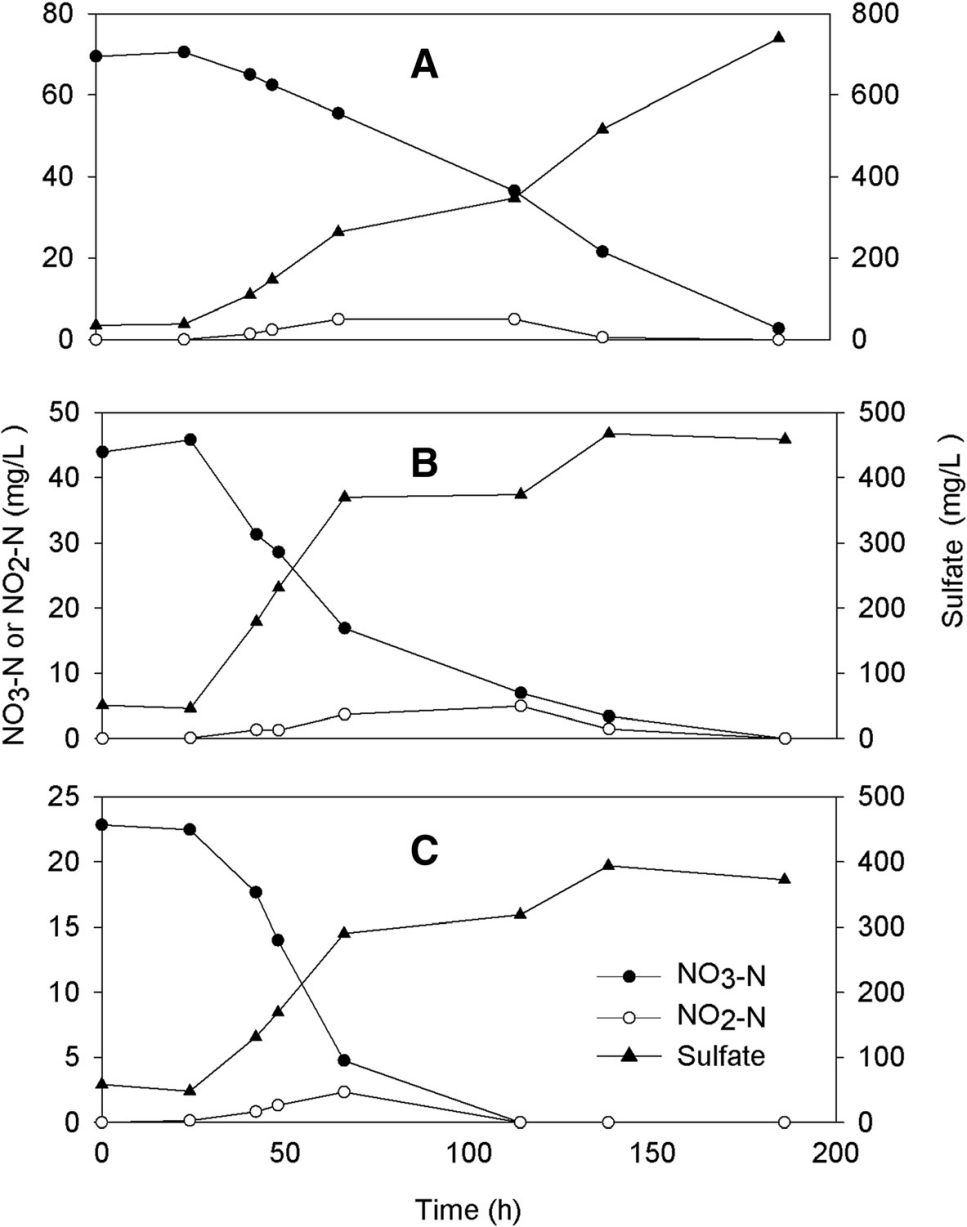
**The variations of NO**_**3**_^**-**^**-N, NO**_**2**_^**-**^**-N and sulfate concentrations in batch experiments at different initial NO**_**3**_^**-**^**-N concentrations [500 μg/L BrO**_**3**_^**-**^**]. (A)** 75 mg/L NO_3_^-^-N; **(B)** 50 mg/L NO_3_^-^-N; **(C)** 25 mg/L NO_3_^-^-N.

Although sulfur-based autotrophic denitrification has several advantages, its main disadvantages are sulfate and acid formation. It is found that sulfate formation was increased as a result of sulfur oxidizing autotrophic denitrification during the experiment (Table 3 and Figures 1 and 2). The concentration of sulfate in autotrophic batch reactor was higher than sulfate limit value of 250 mg/L, set by US EPA.

Figure 2 shows nitrate depletion and sulfate production with time using 500 μg/L bromate and nitrate at three different dosages (25, 50 and 75 mg/L NO_3_^-^-N). The measured maximum NO_2_^-^-N concentrations were 2.3 ± 1.1, 4.9 ± 2.1 and 5.0 ± 2.3 mg/L, respectively (Table 3). Also, produced sulfate concentrations were determined 372, 458 and 749 mg/L in batch reactors for nitrate concentrations of 25, 50 and 75 mg/L respectively. In this study, nitrate reduction rate increased with increasing initial nitrate concentration with transient nitrite accumulation. Similar finding was also reported by Hijnen *et al.*[11].

**Figure 3:**
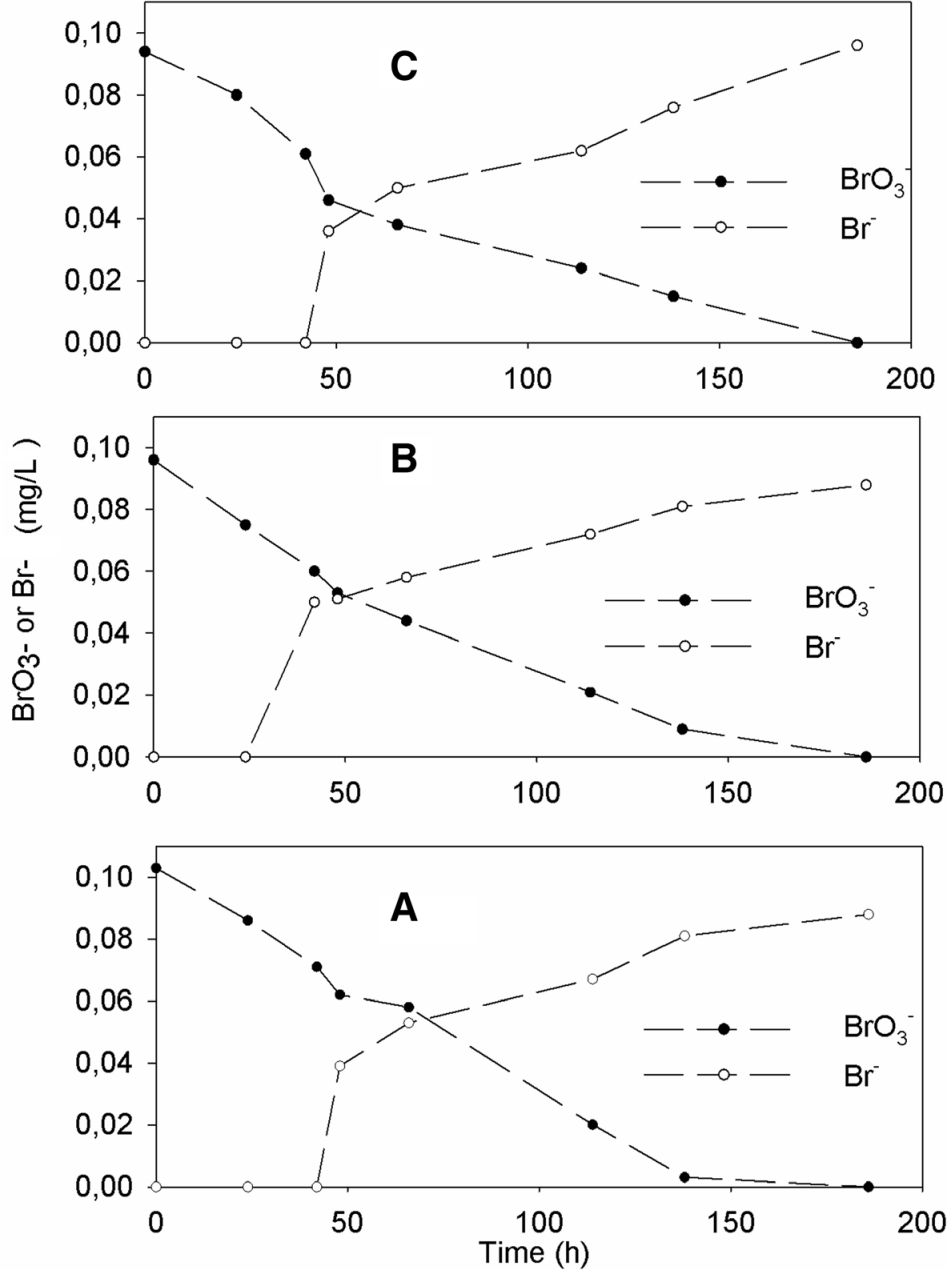
**The variations of BrO**_**3**_^**- **^**in batch experiments at different initial NO**_**3**_^**-**^**-N concentrations [100 μg/L BrO**_**3**_^**-**^**]. (A)** 25 mg/L NO_3_^-^-N; **(B)** 50 mg/L NO_3_^-^-N; **(C)**: 75 mg/L NO_3_^-^-N.

**Figure 4:**
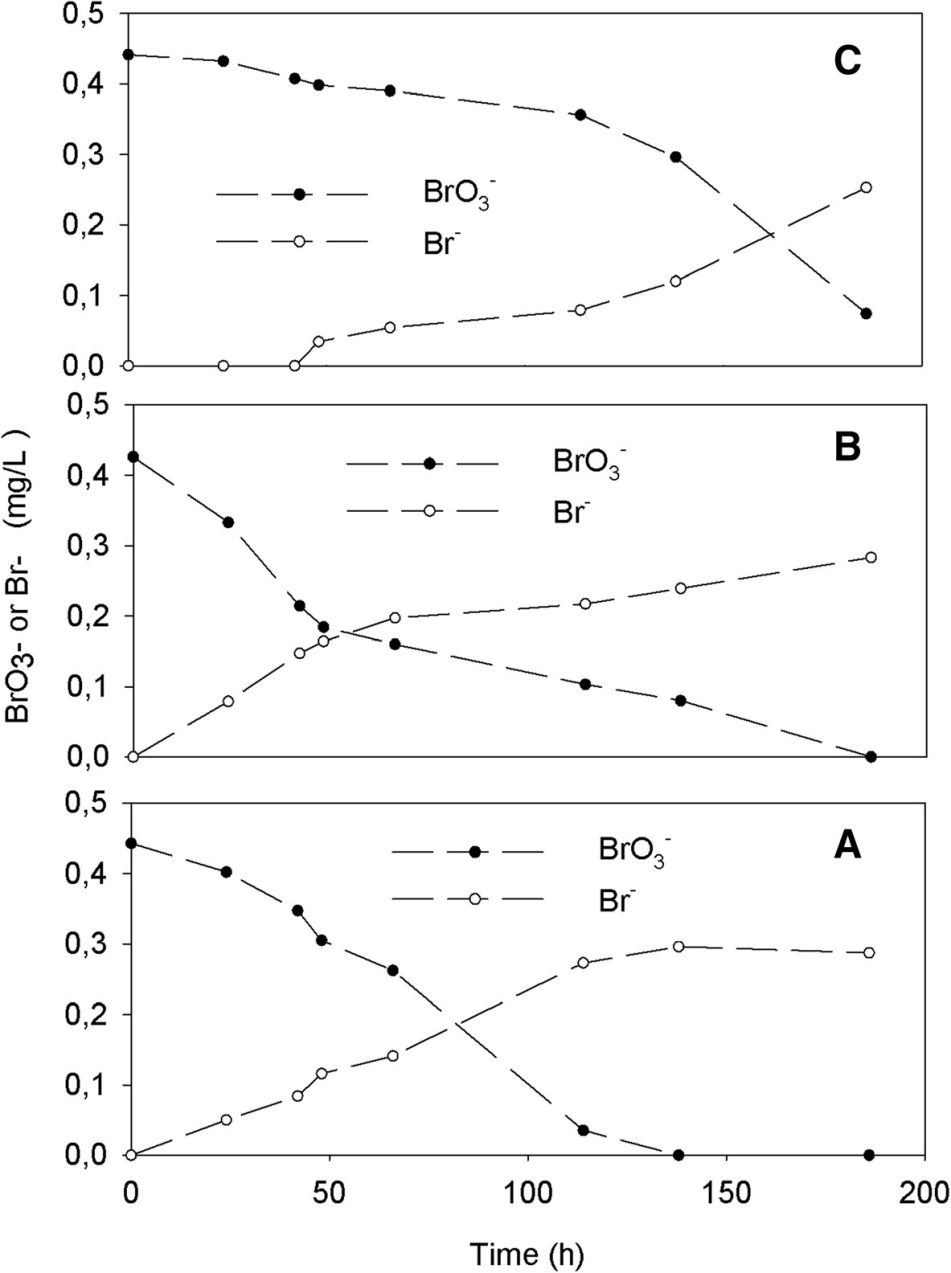
**The variations of BrO**_**3**_^**- **^**in batch experiments at different initial NO**_**3**_^**-**^**-N concentrations [500 μg/L BrO**_**3**_^**-**^**]. (A)** 25 mg/L NO_3_^-^-N; **(B)** 50 mg/L NO_3_^-^-N; **(C)**: 75 mg/L NO_3_^-^-N.

The nitrite accumulation was only transient and complete nitrate denitrification and bromate removal was observed at autotrophic batch experiment. The results of study showed that the bromate removal via autotrophic denitrification process was not effected increasing initial bromate concentrations from 100 to 500 μg/L. Throughout the experiment, decreasing bromate simultaneously with a stoichiometric increase in bromide was observed at the steady state conditions (Figures 3 and 4).

Similar to this study, Krisits *et al.*[8] reported that nitrate reduction rate was faster than bromate. Matos *et al.*[12] noted that bromate reduction is thermodynamically less favorable (it releases less energy) compared to nitrate. Additionally, they observed that increasing initial bromate concentration (from 200 μg/L to 20.48 mg/L) inhibit biological denitrification. The results showed that denitrification rate was relatively lower when increased initial bromate concentration in this study (Table 3). However, complete nitrate and bromate reduction were occurred at the end of the experiments.

### Performance of heterotrophic denitrification process

Heterotrophic denitrifying bacteria use organic compounds as carbon and energy source. The organic compounds are required for complete denitrification depends on the nitrate concentration and bacteria yield coefficient. The nitrite accumulates in water when added organic is stoichiometrically insufficient [6,13]. On the other hand, the residual organic compound remains in the treated water when the added organic is excessive [13].

**Figure 5:**
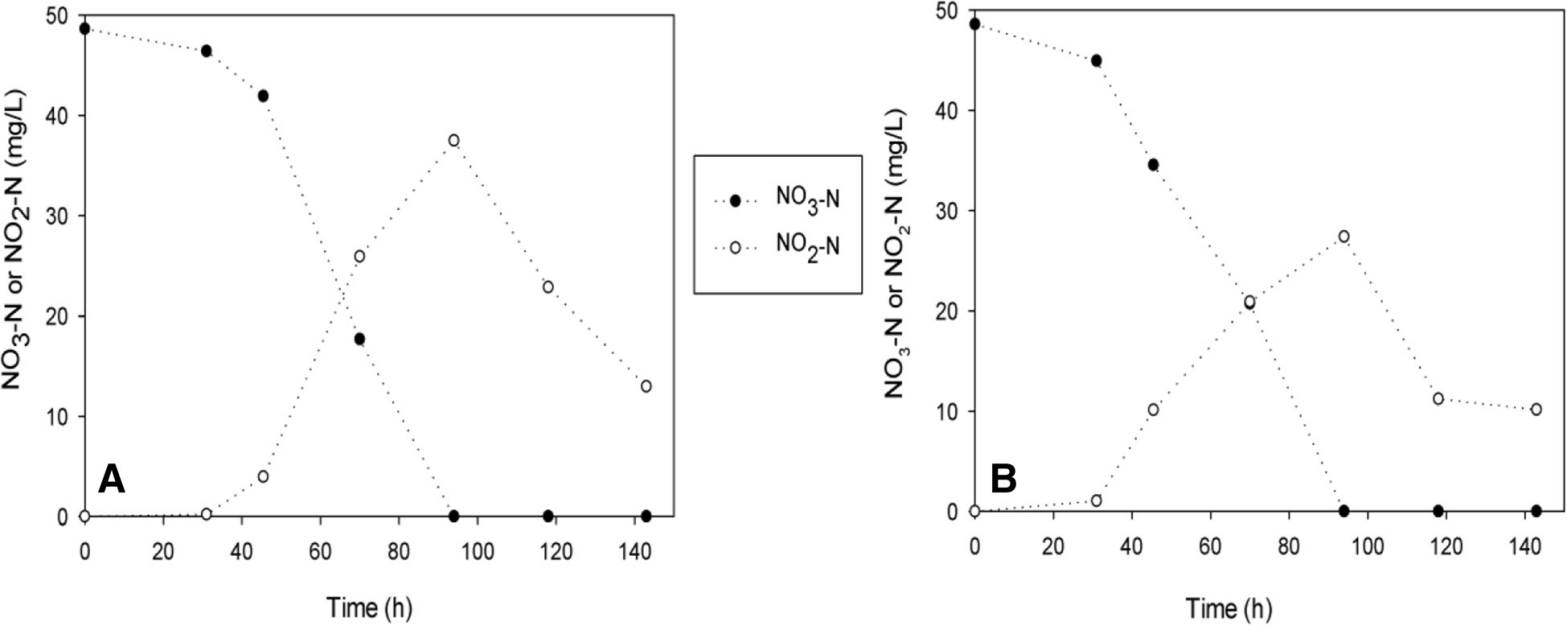
**The variations of NO**_**3**_^**-**^**-N and NO**_**2**_^**-**^**-N in heterotrophic batch experiments at different initial BrO**_**3**_^**- **^**concentrations [19,7 mg/L methanol]. (A)** 100 μg/L BrO_3_^-^**(B)** 500 μg/L BrO_3_^-^.

The C:N ratio is an important factor effecting the performance of heterotrophic denitrification process [14]. Liu *et al.*[15] reported that stoichiometrically C:N (mg CH_3_OH: NO_3_^-^-N) ratio was 2.47 for complete denitrification. In this study, 50 mg/L methanol (mg CH_3_OH: NO_3_^-^-N = 1.11 ˂ 2.47) was added to the R1 and R2 (Table 2). This amount of methanol was lower than the stoichiometric ratio of 2.47 for complete heterotrophic denitrification (Figure 5).

In the presence of 50 mg/L methanol, the nitrite accumulation was observed due to insufficient organic carbon (Figure 5).

**Figure 6:**
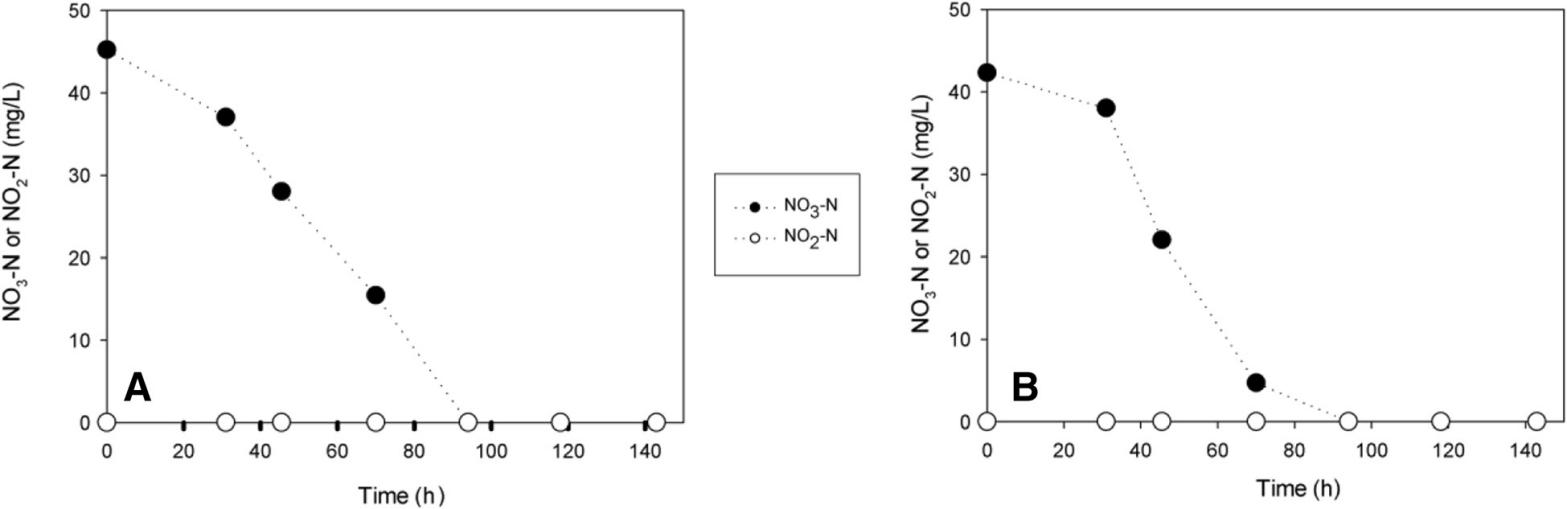
**The variations of NO**_**3**_^**-**^**-N and NO**_**2**_^**-**^**-N in heterotrophic batch experiments at different initial BrO**_**3**_^**- **^**concentrations [45,3 mg/L methanol]. (A)** 100 μg/L BrO_3_^-^**(B)** 500 μg/L BrO_3_^-^.

115 mg/L methanol was added to the R3 and R4 to achieve complete denitrification process. Therefore, the denitrification rate increased with increasing feed methanol concentration as expected (Figure 6). The results with both nitrate and bromate suggest that denitrifying bacteria may be reducing bromate cometabolically.

**Figure 7:**
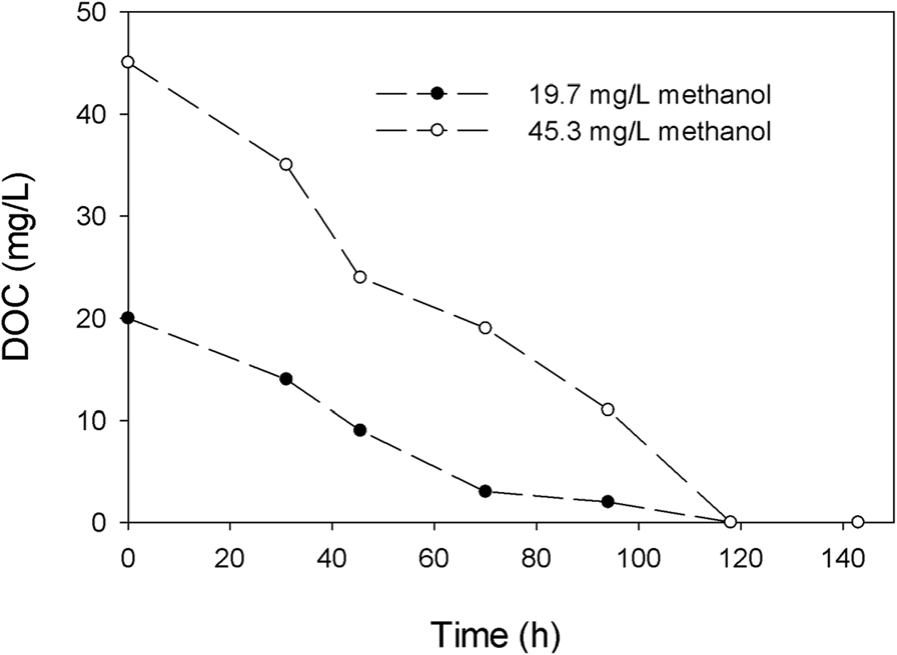
The variations of DOC in heterotrophic batch experiments.

The main disadvantage of the heterotrophic denitrification is needed external carbon resulting in secondary pollution [16]. On the basis of experimental results, the dissolved organic carbon (methanol) was almost completely removed in batch reactors which mean there was no observation of secondary pollution as illustrated in Figure 7. It was favorable for bromate and nitrate removal, especially drinking water treatment.

The exact bromate reduction mechanism by mixed denitrifying population is not known. It seems that bromate is cometabolically reduced by denitrifiers in the presense of nitrate.

## Conclusions

Experiments showed that the denitrification rate was increased with increasing initial NO_3_^-^-N concentration. As a result of sulfur-based autotrophic denitrification, effluent SO_4_^2-^ concentration increased at the end of the process. Effluent bromide measurements indicated that bromate was completely reduced without accumulation of by-products. Additionally, the effect of methanol concentration on bromate reduction by heterotrophic denitrification process was investigated. Batch studies show that NO_2_^-^-N accumulation was observed when methanol was added at methanol/NO_3_^-^-N ratio which was lower than the stoichiometric value (mg CH_3_OH: NO_3_^-^-N = 1.11 ˂ 2.47). This study showed that denitrification process was much faster and nitrite accumulation was not observed when adequate methanol was added. This study can be a contribution for the development of a biological process to remove the bromate and nitrate pollution from drinking water.

## Competing interests

The authors declare that they have no competing interests.

## Authors’ contributions

Corresponding author (SD) carried out design of the study, performed the batch experiments and drafted the manuscript. IB participated in batch reactor design. All authors read and approved the final manuscript.
